# Novel Hybrid Quantum Architecture-Based Lung Cancer Detection Using Chest Radiograph and Computerized Tomography Images

**DOI:** 10.3390/bioengineering11080799

**Published:** 2024-08-07

**Authors:** Jason Elroy Martis, Sannidhan M S, Balasubramani R, A. M. Mutawa, M. Murugappan

**Affiliations:** 1 Department of ISE, NMAM Institute of Technology, Nitte Deemed to be University, Udupi 574110, Karnataka, Indiabalasubramani.r@nitte.edu.in (B.R.); 2 Department of CSE, NMAM Institute of Technology, Nitte Deemed to be University, Udupi 574110, Karnataka, India; sannidhan@nitte.edu.in; 3 Computer Engineering Department, College of Engineering and Petroleum, Kuwait University, Safat 13060, Kuwait; 4 Computer Sciences Department, University of Hamburg, 22527 Hamburg, Germany; 5 Intelligent Signal Processing (ISP) Research Lab, Department of Electronics and Communication Engineering, Kuwait College of Science and Technology, Block 4, Doha 13133, Kuwait; 6 Department of Electronics and Communication Engineering, School of Engineering, Vels Institute of Sciences, Technology, and Advanced Studies, Chennai 600117, Tamil Nadu, India; 7 Center of Excellence for Unmanned Aerial Systems (CoEUAS), Universiti Malaysia Perlis, Arau 02600, Malaysia

**Keywords:** lung tumor classification, deep learning models, quantum layers, transfer learning models, hybrid quantum layer

## Abstract

Lung cancer, the second most common type of cancer worldwide, presents significant health challenges. Detecting this disease early is essential for improving patient outcomes and simplifying treatment. In this study, we propose a hybrid framework that combines deep learning (DL) with quantum computing to enhance the accuracy of lung cancer detection using chest radiographs (CXR) and computerized tomography (CT) images. Our system utilizes pre-trained models for feature extraction and quantum circuits for classification, achieving state-of-the-art performance in various metrics. Not only does our system achieve an overall accuracy of 92.12%, it also excels in other crucial performance measures, such as sensitivity (94%), specificity (90%), F1-score (93%), and precision (92%). These results demonstrate that our hybrid approach can more accurately identify lung cancer signatures compared to traditional methods. Moreover, the incorporation of quantum computing enhances processing speed and scalability, making our system a promising tool for early lung cancer screening and diagnosis. By leveraging the strengths of quantum computing, our approach surpasses traditional methods in terms of speed, accuracy, and efficiency. This study highlights the potential of hybrid computational technologies to transform early cancer detection, paving the way for wider clinical applications and improved patient care outcomes.

## 1. Introduction

The lung is a vital organ for human health, and lung tumors, whether benign or malignant, pose a significant threat by affecting its function and structure. Various causes and symptoms of lung tumors have been identified and reported in the standard research materials. Conducting research on lung tumors is crucial to understanding their mechanisms, diagnosis, treatment, and prevention. The early detection and diagnosis of lung tumors are essential, as they can benefit patients, healthcare systems, and society by minimizing healthcare costs and the complications associated with advanced lung cancer and palliative care. Early intervention can enhance patients’ quality of life, reduce morbidity and mortality, and improve survival chances before the tumor spreads or becomes resistant to treatment.

Computerized tomography (CT) scans are valuable tools for detecting lung cancer, especially in high-risk populations, such as smokers. CT scans provide detailed cross-sectional images of the lungs, allowing for better visualization and assessment of abnormalities compared to chest X-rays (CXR). However, lung tumors can sometimes be visible on CXR but are not clearly detectable on CT scans. This discrepancy may be due to several factors: (1) smaller tumors may be more visible on CXR than on CT scans; (2) the tumor’s location in the lung might affect its visibility, with CXR potentially showing tumors obscured or overlapping with normal lung tissue on CT scans more clearly; (3) each imaging technique has its strengths and weaknesses. Therefore, using both CXR and CT scans is complementary and influential in clinical diagnosis, particularly in lung cancer detection [1,2].

The process of manually identifying tumors is challenging, error-prone, and inconsistent [3]. Depending on the expertise of the radiologist and the prominence of the imaging technique, the response of the radiologist in identifying the tumor will vary. It is possible to identify tumors from various images more quickly, objectively, and precisely by using automated methods, especially deep learning (DL) models [3,4,5]. DL is an advanced tool in artificial intelligence (AI) that uses neural networks to learn from input data and perform tasks such as detection/classification/prediction. In medical imaging, such as CT and CXR, DL techniques have been used to classify lung tumors [6]. The classification of lung tumors is a challenging task that requires the accurate and reliable diagnosis of different types and subtypes of lung cancer, such as medium cell lung cancer and small cell lung cancer. In addition, classifying lung tumors requires a distinction to be made between benign nodules and other lung diseases. Through DL techniques, lung tumor classification can be improved by mining significant features from the input images (CT/CXR), developing robust and efficient DL models, improving performance and interpretability, and providing clinicians with decision support and guidance. By providing complementary information and perspectives about lung anatomy and pathology, CT and CXR images can enhance the accuracy of lung tumor classification. CT images can reveal small lumps not visible on CXRs, providing detailed cross-sectional views of the lungs. While CXRs offer a broader overview of the lungs’ overall geometry, their resolution and projection can sometimes make abnormalities distinctly visible. By combining these imaging modalities, DL techniques can leverage the strengths of both to enhance the reliability of lung tumor classification [6,7].

In general, DL networks require substantial computing power and extended computation times to process data, with performance closely tied to the size of the data and the precision of network hyperparameters. Misconfigured hyperparameters can significantly diminish a model’s accuracy, reliability, robustness, and efficiency. Recent advances in quantum computing offer solutions to these challenges, enhancing the speed, accuracy, and scalability of DL models. By efficiently allocating computation resources, these methods not only accelerate processing speeds but also bolster the robustness and diagnostic accuracy of DL systems. Quantum computing leverages principles, such as superposition, entanglement, and interference to refine classification accuracy. The integration of quantum layers—such as parameterized quantum circuits (PQCs), which can be trained via classical or quantum optimization algorithms—introduces a novel component to traditional networks. These layers have been shown to outperform classical counterparts in various tasks across different datasets, including digital recognition on the Modified National Institute of Standards and Technology (MNIST) database, breast cancer diagnosis, and phase transition detection [8,9]. With ongoing advancements, quantum layers are poised to play a crucial role in the evolution of quantum machine learning and artificial intelligence [10].

In this study, we aim to overcome the shortcomings of existing methods for differentiating benign from malignant lung tumors. CT scans or CXR radiographs are currently used to diagnose lung tumors, but neither provides a comprehensive understanding of the complexity and diversity of these tumors. Additionally, existing methods use DL models that require extensive feature engineering and parameter tuning. Our framework leverages pre-trained transfer learning (TL) models that are fine-tuned for lung tumor classification based on CXR and CT images. In addition, we incorporate a hybrid quantum layer that enhances classification performance by combining CT and CXR features. We evaluate our framework using two standard open-source datasets: ChestX-ray8 and the Lung Image Database Consortium image collection (LIDC-IDRI), which are extensively used in research. The proposed RepVGG model with the hybrid quantum layer achieves a noticeable classification accuracy of over 92%, which is more than 3% higher than other standard methods. 

This research work includes the following contributions to the design of the proposed system:

- There is a new framework proposed for lung tumor classification. It leverages pre-trained TL models that have been fine-tuned for lung tumor classification and uses both CXR and CT images as inputs.
- Hybrid quantum layers that combine CT and CXR data and enhance the TL model to improve classification are introduced.
- The proposed system has been evaluated on two standard datasets and has achieved state-of-the-art performance for lung tumor classification.
- The framework performs better than other methods which rely on either CXR or CT images alone or conventional machine learning methods.

This article is organized as follows: Section 1 introduces the research topic, reviews the existing methods for lung cancer detection and classification, and states the research questions. Section 2 presents a literature review related to the aims and objectives of the proposed system. The methodology of the proposed system is described in Section 3, including pre-processing steps, model architecture, training process, evaluation metrics, and experimental setups. The results of the experiments are presented and analyzed in Section 4, along with comparisons with other state-of-the-art systems and a discussion of the capabilities of the proposed system. Lastly, in Section 5, the article summarizes the major points, presents the novelty and significance of the research, and makes some recommendations for future research.

## 2. State-of-the-Art Research

Many studies have used TL to classify lung nodules or cancers from CT images [11,12,13,14,15,16,17,18,19,20]. TL is a technique that transfers the knowledge acquired from a source domain to a target domain. It can be used to overcome challenges involving limited data in medical image analysis. Different studies have used different convolutional neural network (CNN) architectures and classifiers based on TL, such as VGG16, ResNet50-V2, DenseNet201, SVM, and RF [15,16,17,18,19,20]. The experimental results have demonstrated that TL can enhance the accuracy and performance of lung cancer detection compared to conventional methods [16,17,18]. Wang et al. [16] reported an accuracy improvement of up to 83% for classifying lung cancer, highlighting the effectiveness of TL. Nishio et al. [17] achieved a sensitivity of 82% and specificity of 79%, demonstrating the impact of image size on TL performance. Da Nóbrega et al. [18] also showed that TL could bring the classification accuracy of lung nodules to 85%. Some studies have also investigated the impacts of data augmentation, image size, and ensemble learning on TL [15,17,18,19,20]. The literature review shows that TL is a relevant and effective strategy for lung cancer detection. While most studies focus on applying TL to CT images for lung cancer detection, CXRs are equally important. They are more widely used and accessible, but they pose challenges for TL due to their low quality. However, CT images also have drawbacks [6,7]. Exploring TL for CXR images may require different techniques.

Several studies have used DL techniques for lung disease classification using both CXR and CT images, which can improve the detection of lung abnormalities, such as pneumonia, cancer, and COVID-19. Refs. [21,22,23] utilized different pre-trained CNN models to classify both types of images (CXR and CT scans), achieving high accuracy and reporting better results than other related works in their literature. In addition, the researchers have used a tuned VGG-19 model to detect COVID-19 using features extracted from both types of images, which achieved high accuracy of 81%, 83% sensitivity, and 82% specificity [24]. The review by Shyni et al. [25] further supports the combination of CT and CXR images to provide faster and more accurate results along with data scarcity challenges. Their study reported a notable increase in diagnostic accuracy, where the combined approach achieved an accuracy of approximately 84%. This was a significant improvement over models trained solely on CXR or CT images, which generally achieved accuracies of around 74% and 70%, respectively. Moreover, the sensitivity and specificity of the combined models reached as high as 83% and 85%, respectively, compared to 75% sensitivity and 77% specificity for models using only CXR images, and 69% sensitivity and 70% specificity for those using only CT images.

Quantum computing has been shown to enhance the performance of DL network systems in various applications. QCNN is a novel DL technique that combines quantum and classical computing to process image data. In [26,27], the researchers demonstrated the advantages of QCNN over classic CNN in terms of accuracy and speed on different image classification tasks. In [26], a reported 7% improvement in accuracy was noted, and in [27], accuracy was improved by 10% over traditional CNNs. Both articles also explored the correlation between the chaotic nature of the image and the QCNN performance and found that quantum entanglement plays a key role in improving classification scores. Recently, researchers have proposed a variational quantum deep neural network (VQDNN) model that uses parametrized quantum circuits to achieve greater accuracy improvement of approximately 8% than classical neural networks on two datasets with limited qubits in image recognition [28]. In addition, the authors in [29,30] explore the use of hybrid TL techniques that combine a classical pre-trained network with a variational quantum circuit as the final layer (classifier) on small datasets. They evaluate different classical feature extractors with a quantum circuit as a classifier on three image datasets: trash (recycling material), tuberculosis (TB) from CXR images, and cracks in concrete images. They show that the hybrid models outperform the classical models by demonstrating an improvement in accuracy rate of over 12% on all datasets, even with qubit constraints. In [31], the researchers introduce a new kind of transformational layer for image recognition, called a quantum convolution or quanvolution layer. Quanvolution layers use random quantum circuits to locally transform the input data, similar to classical convolution layers. They compare classical convolutional neural networks (CNNs), quantum convolutional neural networks (QCNNs), and CNNs with extra non-linearities on the MNIST dataset. They show that QCNNs have faster training and higher accuracy improvement of 9% over traditional CNNs, suggesting the potential of quanvolution layers for near-term quantum computing.

A review of the existing literature found that DL techniques can help with the challenging and important task of classifying lung diseases using medical images. Many studies have used TL to achieve better results than conventional methods for classifying lung nodules or cancers from CT/CXR images with different CNN architectures and classifiers. Many studies have also shown that QCNNs can outperform classic CNNs in accuracy for different image classification tasks by increasing the speed of computation, and scalability, and reducing the computation power. Quantum computing can boost the performance of DL network systems in various applications. Some studies have used variational quantum circuits to enhance the performance of QCNNs. Based on these findings, we propose a new system that combines TL and QCNNs for classifying lung diseases using both CXR and CT images. We aim to use quantum computing to improve the performance of TL models for medical image analysis. Table 1 provides the summary of the literature review conducted.

## 3. Methodology

**Figure 1:**
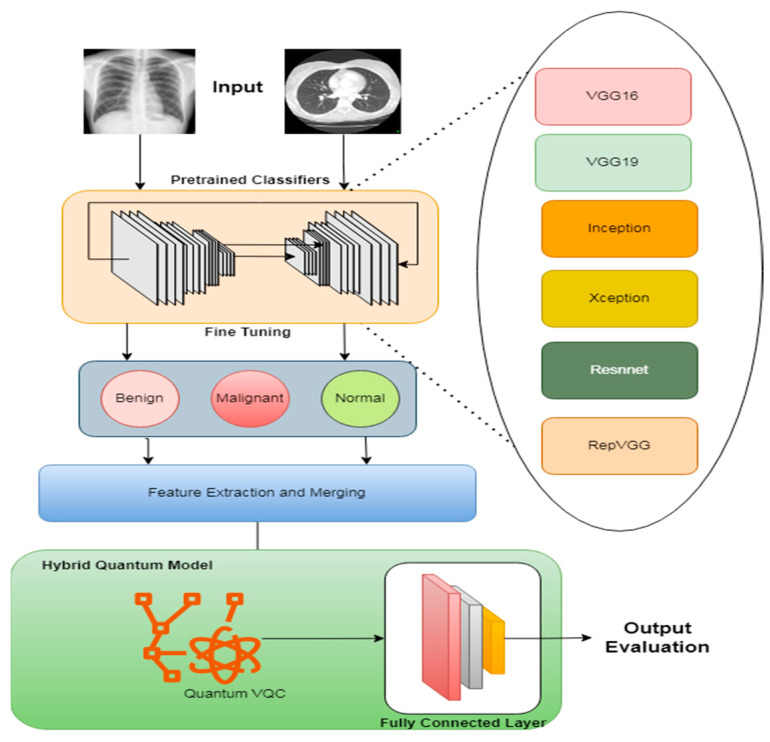
Proposed system’s architecture.

This section outlines a proposed system that integrates TL and QCNNs to enhance lung disease classification using chest X-ray (CXR) and computed tomography (CT) images. The process begins with acquiring and pre-processing extensive medical image datasets to ensure high quality and uniformity. Pre-trained CNN models, such as VGG16, ResNet50-V2, and DenseNet201, are fine-tuned for specific lung disease classification tasks. QCNNs are developed and integrated with these TL models to create a hybrid system that leverages both classical and quantum computing advantages. The hybrid models are trained, optimized, and evaluated to maximize performance metrics like accuracy, sensitivity, and specificity. Finally, the optimized model is prepared for deployment in clinical settings, ensuring scalability and seamless integration with existing medical systems. This approach aims to overcome data limitations and improve the accuracy and efficiency of lung disease detection. Figure 1 illustrates the overall working steps of the proposed system. This approach aims to overcome data limitations and improve the accuracy and efficiency of lung disease detection.

The proposed system, as depicted in Figure 1, has three main modules that work together: (1) image acquisition, (2) tuning of the TL model, and (3) quantum learning and classification. The following subsections describe each module in detail.

### 3.1. Input Image Description

Images are collected from both CXR and CT scans during the image acquisition process. CXR and CT scans are used as the source of the images. The classification task is challenging since CT scans and CXR belong to two different types of images. As a result, we train the network separately for CXR and CT scans, which improves the accuracy and efficiency of feature extraction. Images are converted to grayscale, with a range between 1 and 255. A mathematical formula for the image retrieval process is shown in Equations (1) and (2).

(1) $$I_{x}(x,y)\leftarrow dataset(CXR)$$

(2) $$I_{ct}(x,y)\leftarrow dataset(CT)$$

Here, *I_x_*(*x*, *y*) is the image taken from a dataset of CXR by means of pixels. Similarly, *I_ct_*(*x*, *y*) stands for the images from the CT dataset. The values (*x*, *y*) are generic to represent the width and height of a single image, respectively. It is necessary to resize all images, since neural networks require them to have a fixed size. Nevertheless, resizing has its trade-offs: reducing the size of an image reduces its quality, whereas making it larger increases training time and complexity. To find a balance between computational cost and accuracy, based on experimental investigation, we use 1024 × 1024 pixels as the resized image size [32]. The relevant evidence for this is presented in the experimental trials conducted in Table 2.

### 3.2. Tuning of Transfer Learning Model

The purpose of this process is to categorize CXR and CT images into benign, normal, and malignant groups. Malignant tumors can spread beyond the body and pose a threat to other organs. Benign tumors are harmless growths that do not invade nearby tissues. An organ classified as normal works well and has no tumors. As explained in more detail in the following sections, we use a hybrid quantum model in this paper to classify the images.

#### 3.2.1. Feature Extraction

**Figure 2:**
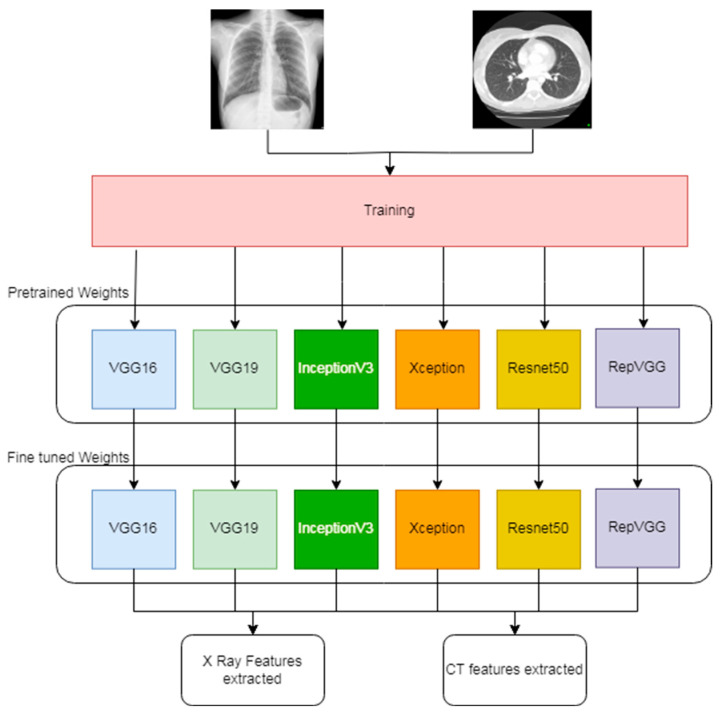
Leveraging transfer learning for feature extraction from CT and CXR images.

Feature extraction is a crucial step in the field of DL. It employs notable structures that enable the system to assess the structures according to their corresponding classes. TL is a quick training approach that hastens the extraction of features and avoids overfitting by manually training the system. TL involves using pre-trained models that are used for other classification jobs. Using the knowledge gained, we can extrapolate it to suit our needs within a minimum training time. Figure 2 shows the architecture describing the internal structure of the TL model adopted for training.

As shown in Figure 2; first, we used pretrained TL models like VGG16, VGG19, Inception-v3, Xception, ResNet50, and RepVGG to extract features [33,34,35]. We chose these models based on their variation in convolutional filter usage and the fact that they were developed for different classification problems. Furthermore, we replaced the top classification layer with our own classification rule. Table 2 presents an overview of various pre-trained CNN models used for feature extraction in our study. Each model was evaluated based on its size, the number of hyperparameters, the specific layer used for feature extraction, the initial feature dimension, and the dimension after fusion.

**Table 2:** Summary of pre-trained models used for feature extraction in our research.

Model Name	Size (MB)	Hyperparameters (Million)	Feature Extraction Layer	Feature Dimension	Dimension after Fusion
VGG16	528	138.35	Block5_conv3	512	1024
VGG19	549	143.66	Block5_conv4	512	1024
InceptionV3	92	23.85	mixed10	2048	4096
Xception	88	22.91	block14_sepconv2_act	2048	4096
ResNet50	99	25.636	conv5_block3_out	2048	4096
RepVGG	558	11.68	repvgg_block5	2048	4096

These pre-trained classifiers are fine-tuned on the CXR and CT datasets separately to obtain optimal models serving to extract features from CXR and CT scans. Equations (3)–(5) explain the structure of how features are extracted and finetuned for our classification purpose.

(3) $$a^{\left(l\right)}=f\left(W^{\left(l\right)}\times x^{\left(l-1\right)}+b^{\left(l\right)}\right)$$

(4) $$\mathrm{ReLU}\left(x\right)=\max\left(0,x\right)$$

(5) $$z=W^{\left(f\right)}*a^{\left(L\right)}+b^{\left(f\right)}$$

**Figure 3:**
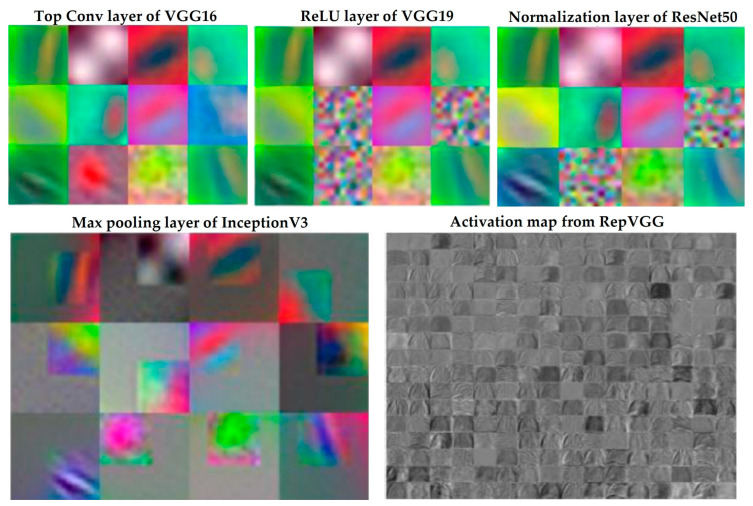
Visual analysis of different layers of TL framework.

Here, $x^{\left(l-1\right)}$ is the input to the layer *l* (for the first layer, *x*^0^ is the input image). *W*^(*l*)^ and *b*^(*l*)^ are the weights and biases of the layer *l*, respectively. *f* is the activation functions which are either ReLU or sigmoid. *a*^(*L*)^ is the output of layer *l* after applying the activation function. *L* is the last pre-trained layer, *W*^(*f*)^ and *b*^(*F*)^ are the weights and biases of the final fully connected layer, and *z* is the logits vector representing the raw model predictions. It is necessary to discard the last layer of each model in order to classify the model into our necessary classes. Finally, the CXR and CT datasets are stored separately because they have distinct feature sets. The following sections elaborate on some sample layers for image classification that incorporate these features. Figure 3 illustrates how features are accessed from selected layers of a proposed TL framework. 

The visualization in Figure 3 showcases how various neural network layers process X-ray and CT scan images, highlighting distinct feature extraction methods for each type of imaging data.

For X-rays, the sequence begins with the top convolutional layer of VGG16, which identifies low-level features, such as edges and textures, essential for delineating anatomical structures. This is followed by the ReLU layer of VGG19, which enhances these features by removing negative values, thus improving the visibility of critical details like lesions or masses. The normalization layer of ResNet50 then adjusts the feature maps to a consistent scale, aiding in uniform feature interpretation across different X-ray images.

In CT scans, the max pooling layer of InceptionV3 reduces spatial resolution but retains significant features within each region, focusing the analysis on relevant aspects, such as tumors. The activation map from RepVGG synthesizes higher-level features, revealing complex tissue textures and enhancing the model’s ability to detect abnormalities. 

#### 3.2.2. Merging of Features

In this study, we utilize both computed tomography (CT) and chest X-ray (CXR) imaging modalities for each scan to maximize the diagnostic potential of the imaging data. Features are independently extracted from both the CT and CXR images to harness the unique diagnostic information each modality provides. The detailed set of procedures is explained as follows:

- Feature Extraction Process:

In this process, the set of features from the CT images uses a dedicated TL model optimized for CT data. These features typically capture detailed anatomical structures and potential abnormalities specific to CT imaging. Equation (6) depicts the mathematical formulation of this process,

(6) $$F^{x}\leftarrow <f_{1}^{x},f_{2}^{x},f_{3}^{x},\;\ldots ,\;f_{n}^{x}>\leftarrow TL(I_{x})$$

Similarly, a different set of features is extracted from the corresponding CXR images using another TL model that is specifically tuned to exploit the diagnostic strengths of CXR, such as overall lung geometry and certain types of lesions more visible in CXR. The extraction process is explained in Equation (7)

(7) $$F^{ct}\leftarrow <f_{1}^{ct},f_{2}^{ct},f_{3}^{ct},\;\ldots ,\;f_{n}^{ct}>\leftarrow TL(I_{ct})$$

- Feature Merging Strategy:

The features extracted from both CT and CXR images are then merged to form a combined feature vector. This merging process involves concatenating the feature vectors from each modality. The process of feature merging is depicted in Equation (8) mathematically.

(8) $$F_{total}\leftarrow F^{x}+F^{ct}\leftarrow \left\{<f_{1}^{x},f_{2}^{x},f_{3}^{x},\;\ldots ,\;f_{n}^{x}>||<f_{1}^{ct},f_{2}^{ct},f_{3}^{ct},\;\ldots ,\;f_{n}^{ct}>\right\}$$

In Equations (6)–(8), $f_{1}^{x}$ represents the single feature obtained from an CXR image. Similarly, $f_{1}^{ct}$ represents a single feature obtained from a CT image. Also, *F^x^* and *F^ct^* represents the feature vector of CXR and CT scans, respectively. *F_total_* stands for a simple concatenation of the features of both *F^x^* and *F^ct^*.

#### 3.2.3. Dimensionality Reduction

This step reduces the dimensionality of the data by applying a layer that transforms many input features into fewer output features. As part of our process, we use a singular value decomposition (SVD) layer to compress the merged input features extracted from the TL models into five quantum features. The main reason for selecting SVD is due to its ability to optimally represent and denoise high-dimensional medical imaging data [36]. The number of features we chose is fixed because it is appropriate for our needs. In Equations (9)–(12), we see the transformation function for singular value decomposition (SVD).

(9) $$U,\sum ,V=SVD(original_{dimensions})$$

(10) $$U=M_{\left[entries\times 5\right]}$$

(11) $$\sum =M_{\left[5\times 5\right]}$$

(12) $$5\times 5=M_{\left[5\times dimensions\right]}$$

Here, *U* represents the complex unitary matrix having a column size of the reduced number of dimensions. ∑ represents a natural number nonnegative diagonal matrix of 5 × 5. *V* stands for a complex unitary matrix of five rows having original dimensional columns. Note that for SVD, we will not be using *V* but *V^T^* which is its transpose.

#### 3.2.4. Quantum Layer

Circuits with variable parameters, known as variational circuits, play an important role in quantum computing. They are analogous to neural networks in classical computing, which are powerful machine learning models [37,38,39]. In this study, we implemented a quantum variational circuit with five qubits, each representing a classical binary bit (0 or 1). Quantum states of electron spin can be determined by qubits in a magnetic field, leading to spin-up (1) or spin-down (0) states. This spin state represents the fundamental binary information in quantum computing, similar to classical bits but with the added advantage of quantum superposition and entanglement.

Our quantum variational circuit is composed of three key states: initial, parameterized, and measurement. In the initial state, all qubits are initialized to 0. This initialization ensures a known starting point for subsequent quantum operations.

In the parameterized state, the quantum circuit receives two types of input parameters: input data and variational parameters. The input data represent the classical information to be processed, while the variational parameters are tunable parameters optimized during the training process to minimize the cost function. The classical data are inserted into these quantum circuits using quantum embeddings, which map classical data into high-dimensional Hilbert space, enabling the quantum circuit to process it. The final state is the measurement state, where the quantum system is measured, and the resulting quantum states are collapsed into classical binary outcomes (0 or 1). The measurement results are used to evaluate the performance of the quantum circuit and adjust the variational parameters accordingly.

**Figure 4:**
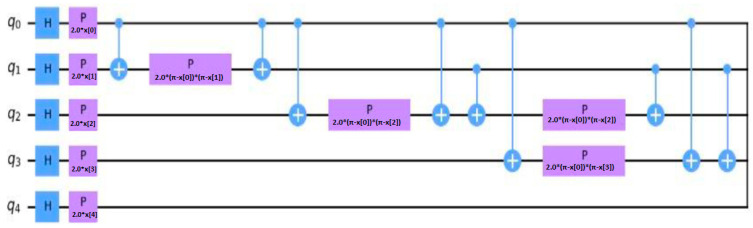
The architecture of the quantum variational circuit with five qubits.

Our quantum variational circuit architecture, as illustrated in Figure 4, integrates these three states into a cohesive framework. The figure provides a visual representation of the quantum circuit, detailing the flow of information from initialization through parameterization to measurement. This architecture leverages the principles of quantum mechanics to perform complex computations, offering the potential for significant advancements in computational power and efficiency compared to classical methods. Classical data integration into quantum circuits is facilitated by quantum embeddings, which utilize Hilbert spaces for feature mapping. This approach allows the quantum variational circuit to process classical data within the quantum domain, harnessing the unique computational capabilities of quantum mechanics.

Figure 4 illustrates the architecture of our proposed quantum circuit, detailing the initialization of qubits, the parameterization process, and the measurement outcomes. This comprehensive illustration underscores the intricate design and operational flow of the quantum variational circuit implemented in this study.

In Figure 4, H represents a Hadamard gate. P, also known as the phase gate or phase shift gate or S gate, is also a single-qubit operation. It changes the phase of a spin along a specific axis. The Hadamard gate is a single-qubit operation that maps the basis state |├ 0⟩ to (|├ 0⟩ + |├ 1⟩)/√2 and |├ 1⟩ to (|├ 0⟩ − |├ 1⟩)/√2. The equations concerning the Hadamard gate and the P gate are shown in Equations (13) and (14), respectively [40].

(13) $$H=\frac{1}{\sqrt{2}}\left(\begin{array}{cc} 1 & 1\\ 1 & -1 \end{array}\right)$$

(14) $$S=\left(\begin{array}{cc} 1 & 0\\ 0 & i \end{array}\right)$$

#### 3.2.5. Fully Connected Layer

**Figure 5:**
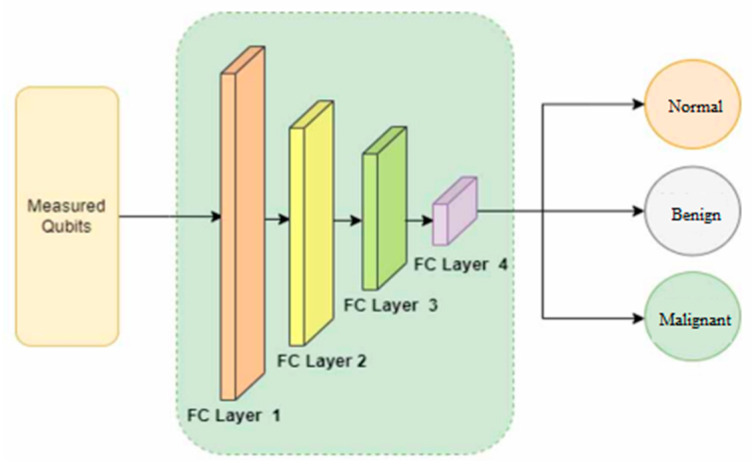
The QCNN architecture with quantum operations and measurements.

A fully connected layer is one in which each neuron in one layer connects to every neuron in another layer. Most often, it is the last layer in a network that produces output. In hybrid quantum networks, a fully connected layer can be achieved by using quantum operations, such as controlled-NOT gates, Hadamard gates, and measurements [41,42]. Quantum operations are unitary matrices that transform the quantum state of neurons. The measurement of a quantum state on a specific basis can provide the output of a quantum operation. It is a network architecture that allows any two users to share entanglement resources and perform quantum distribution without trusting any nodes [43]. In a fully connected quantum network, multiple users can communicate in a highly secure and efficient manner. With QCNN, we leverage quantum advantages, such as superposition and entanglement, to extend the capabilities of classical CNNs. In QCNNs, three layers are present: quantum convolutional layers, pooling layers, and fully connected layers [44,45,46]. In the quantum convolutional layer, data are filtered using a quantum filter mask, and a new quantum state is generated. A coarse-graining operation is performed on the pooling layer to reduce the dimensionality of the data. In the fully connected layer, quantum operations and measurements are used to calculate the final output. Figure 5 graphically illustrates our proposed architecture as it relates to measured qubits. Four layers, each made up of hundred, fifty, twenty, and three neurons, are used in our fully connected layer to aid image classification.

## 4. Experimental Results and Discussion

In this section, we conduct various analyses to evaluate the performance of our hybrid quantum model. In each subsection, we present the results of different analyses.

### 4.1. Dataset Description

Two primary datasets are used in this study: ChestX-ray8 and LIDC-IDRI [47]. There are fifteen classes of chest CXR in ChestX-ray8, some of which are benign, others malignant, and some are normal. The images are 1024 × 1024 pixels and there are 112,120 images in total. There is a variety of different sizes of nodules in the LIDC-IDRI dataset, which was acquired from clinically acquired CT images of the lungs. A total of 1018 slices were obtained from 1010 lung CT scans. This study used a subset of 5000 lung scans that covered nodules and regions without nodules to ensure comprehensive coverage and representativeness. This subset includes malignant (1000 images), benign (500 images), and normal (500 images). Preprocessing steps included normalization, resizing all images to a consistent resolution, and data augmentation techniques, such as rotation, flipping, and scaling, to increase diversity and prevent overfitting. Poor-quality images or those with artifacts were removed. Inclusion criteria were clear labeling for ChestX-ray8 images and clear annotations for LIDC-IDRI scans. Exclusion criteria included ambiguous labels and low-quality scans. Table 3 presents a brief overview of the datasets after filtering out elements suited to our study. 

#### Visual Presentation of the Dataset Images

**Figure 6:**
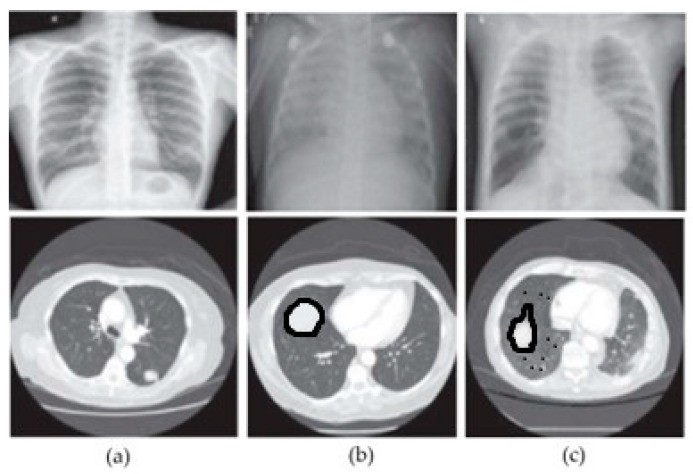
Sample images from the adopted datasets. (**a**) Normal, (**b**) benign, (**c**) malignant.

In this section, we show examples from each of the three classes that we used in our study in order to illustrate the variety of images in the dataset. Figure 6 shows a selection of images from both datasets, representing different classes. The first column shows images from the normal class; the second column shows images from the benign class; and the third column shows images from the malignant class. Similarly, the first row represents CXR images corresponding to each class, while the second row represents CT images corresponding to each class.

Based on the analysis of Figure 6, we can visually observe a slight similarity between the images indicating a particular pattern. Hence, merging features can improve a machine’s classification accuracy.

### 4.2. Analysis Concerning Image Size vs. Computational Cost

In this section under Table 4, we describe the resource requirements for classifying lung samples based on image size. A set of three sizes is used, such as 1024 × 1024, 448 × 448, and 224 × 224. The smaller the image size, the fewer resources are needed. In addition, the third row (224 × 224) of the table has a large variance, resulting in less training time but a lower accuracy rate. The first two variants, however, have a reasonable amount of accuracy with a difference of −2%, which is acceptable given the difference in training time.

### 4.3. Per Epoch Accuracy Analysis

We ran our proposed architecture on a DL server on a dual Intel Xeon E5-2609V5 Tesla NVIDIA P100 GPU with a total of 3585 cores clocked at a maximum speed of 18.9 Teraflops and listed its different epochs. The system has a RAM capability of 128 GB running Ubuntu 18.04 LTS. We used Keras as our framework, which runs on TensorFlow 2.10. Since the system ran on 550 epochs, Table 5 shows brief accuracy and loss values during specific epoch intervals of a certain hybrid quantum model containing RepVGG. The parameters chosen were training accuracy and training loss.

**Figure 7:**
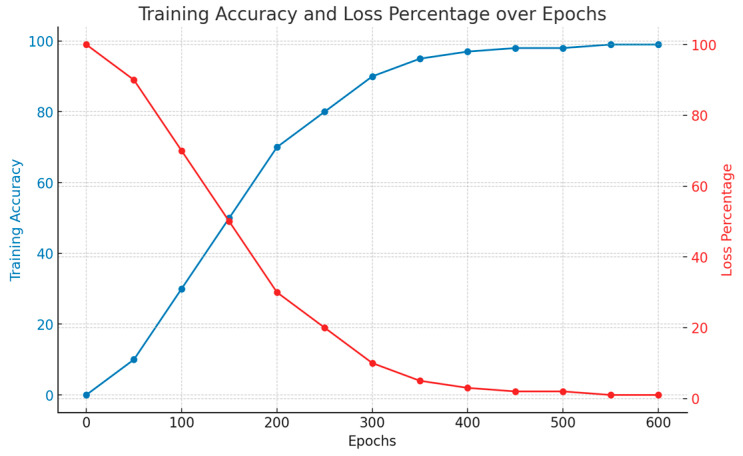
Training and loss accuracy for different epochs of the system.

According to Table 5, a maximum accuracy of 92.12% was reached at epochs 500 with a loss percentage of 7.88%. A certain hybrid quantum model containing RepVGG showed brief accuracy and loss values during specific epoch intervals. Training accuracy and training loss were chosen as parameters. The plot in Figure 7 shows that the data are neither over-fitted nor under-fitted, as the training accuracy curve in Figure 7 follows a typical learning pattern. Likewise, the loss curve in Figure 7 shows a normal decrease as the epochs increased.

### 4.4. Analysis Concerning Accuracy with and without Quantum Models

Comparing the performance of the system with and without a quantum classifier was conducted to demonstrate the effectiveness of the proposed architecture. A comparative analysis of the system without quantum classifier (traditional) versus with quantum classifier (hybrid) is presented in Table 6 [48,49,50].

Based on the data in Table 6, our hybrid quantum system improves the overall accuracy of the system, with RepVGG leading the way with an overall rate of 92.12%. The results of this study indicate that quantum systems have an added benefit over traditional DL systems. In addition, the marginal split of all the models’ misclassifications with and without the quantum system is shown in Table 7 [21]. 

**Figure 8:**
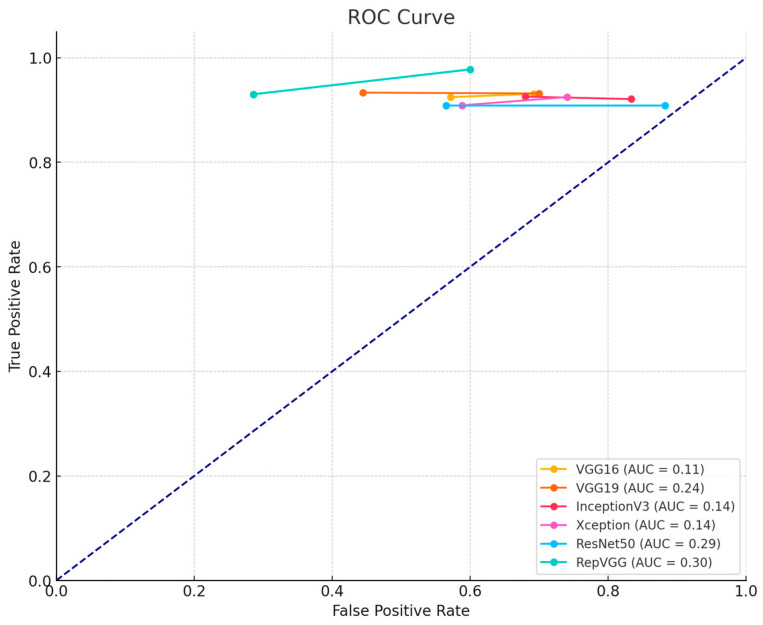
Performance evaluation of hybrid models using ROC curves.

**Figure 9:**
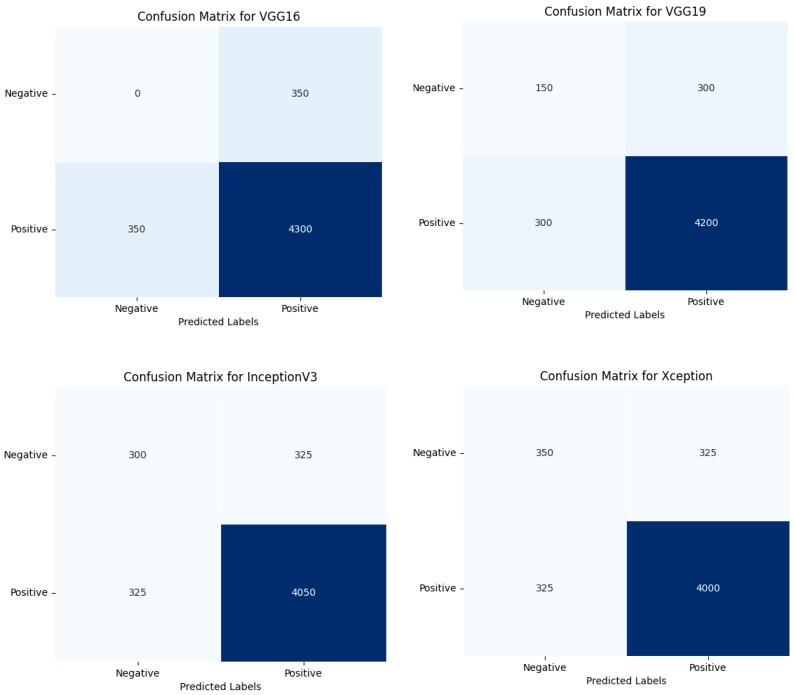

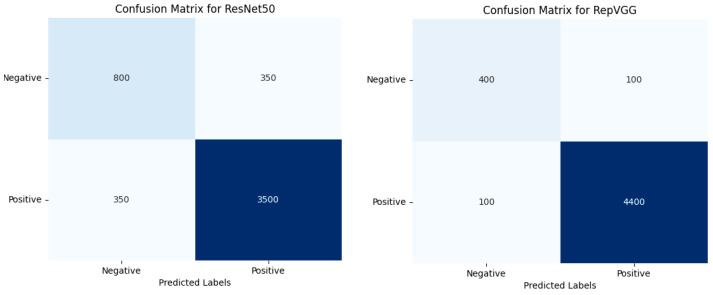
Performance evaluation of hybrid models using confusion matrices.

We also plotted the performance of each hybrid model used in our study through receiver operating characteristic (ROC) curves and confusion matrices. These visualizations provide a deeper insight into the effectiveness of each model used. The ROC plot is presented in Figure 8 and the confusion matrix is presented in Figure 9.

The ROC curves illustrate the true positive rate (sensitivity) against the false positive rate (1-specificity) for various threshold settings. A higher area under the curve (AUC) indicates better performance in distinguishing between classes. The ROC curves for our hybrid models demonstrate their superior ability to accurately classify lung tumor images, showcasing the benefits of integrating quantum computing with traditional deep learning methods.

Figure 9’s confusion matrices highlight the superior performance of our hybrid models, showing high true positives (TP) and true negatives (TN) while minimizing false positives (FP) and false negatives (FN). This indicates improved accuracy, precision, and recall compared to traditional models. The hybrid models, especially RepVGG with quantum layers, demonstrate significant diagnostic improvements.

### 4.5. Comparision between Merging and Not Merging of Features

Table 8 summarizes the classification accuracy achieved by using features from individual models without merging and the improved accuracy obtained by merging features from different models. It also includes the feature dimensions before and after fusion.

Table 8 demonstrates that merging features from different TL models significantly improves classification accuracy. This improvement across all models validates that merging features captures more detailed patterns, enhancing data representation and classification performance.

### 4.6. State-of-the-Art Comparison

We have also evaluated the classification performance and strength of our hybrid quantum system against other existing state-of-the-art systems. A comprehensive comparison of our system with classification systems and traditional quantum systems is presented in this paper. The Table 9 shows the overall performance of the system.

Based on the data presented in Table 9, our hybrid quantum system appears to perform better in terms of accuracy level and training time. As a result, our system performed better across the board, proving the strength of our proposed architecture in all areas.

## 5. Conclusions

In this paper, we propose a new framework for lung tumor classification that uses both CT and CXR images as inputs and pre-trained TL models that are tailored to this task. The TL model has been improved by combining features learned from CT and CXR images with a hybrid quantum layer. On two standard datasets, ChestX-ray8 and LIDC-IDRI, we successfully classified lung tumors using our framework. In addition to our framework, other techniques relying on CXR or CT images alone or on conventional machine learning models do not achieve the same results. We demonstrate that lung tumor classification can be improved using both imaging modalities and quantum computing. As a result, early detection, treatment, and outcome of lung cancer patients can be greatly improved. 

It is important to note that the following are some possible limitations of the work in relation to the conclusion of the paper:

- There may be some types of lung cancer that are not suitable for the framework because of their distinct morphological or molecular characteristics.
- It should be noted that the framework may not capture the diversity and intricacy of lung tumor staging, which may have a substantial impact on the patient’s outcome and management.
- In settings with limited resources, the framework may be inaccessible or expensive, especially in situations where resources are limited.
- We tested the proposed model with a small number of images taken from two different datasets. Nevertheless, the proposed framework needs to be standardized by testing it against a larger number of unknown or new data sets.
- This study focuses solely on non-invasive imaging techniques and excludes biopsy, the definitive method for lung cancer diagnosis. While this approach reduces patient risk, it may not capture the comprehensive accuracy provided by biopsy. Future research could integrate these methods to enhance both early detection and diagnostic confirmation.

We are planning on applying our model to other types of lung diseases as well as other imaging methods in the future. Furthermore, to further improve our framework’s performance, we can experiment with other quantum layers and optimization methods in order to further improve the performance of our framework.

## Figures and Tables

**Table 1:** Literature review summary.

Reference	Approach	Key Findings	Identified Gaps
[11,12,13,14,15,16,17,18,19,20]	TL	TL enhances accuracy and performance for lung cancer detection. Different CNN architectures and classifiers used, such as VGG16, ResNet50-V2, DenseNet201, SVM, and RF.	Limited data availability in medical image analysis. Need for techniques for CXR.
[16]	TL	Accuracy improvement for lung cancer classification. Reported accuracy up to 83%.	Impact of image size on TL performance not fully explored.
[17]	TL	Demonstrated the impact of image size on TL performance. Sensitivity 82%, specificity 79%.	Need for optimization of TL models for different image sizes.
[18]	TL	Enhanced classification accuracy of lung nodules. Accuracy up to 85%.	Requires further validation on larger datasets.
[21,22,23]	DL	High accuracy for lung disease classification from CXR and CT images. Achieved higher accuracy than other related works.	Integration of pre-processing and augmentation techniques needs further exploration.
[24]	DL	COVID-19 detection from CXR and CT images. Accuracy 81%, sensitivity 83%, specificity 82%.	Limited by data scarcity and need for larger, diverse datasets.
[25]	DL	Combined CT and CXR approach for COVID-19 diagnosis: accuracy 84%, sensitivity 83%, specificity 85%.	Challenges in combining different image modalities for consistent performance.
[27]	QCNN	Correlation between image chaos and QCNN performance. Reported a 10% accuracy improvement.	Understanding the role of quantum entanglement in performance improvement.
[28]	VQDNN	Better accuracy improvement on limited qubit datasets. Reported 8% accuracy improvement.	Qubit limitations and practical implementation challenges.
[29,30]	Hybrid TL	Improved accuracy with small datasets. Over 12% accuracy improvement.	Need for more extensive testing across different types of datasets.
[31]	Quanvolution Layer	Faster training and higher accuracy on MNIST. Reported 9% accuracy improvement.	Integration with classical CNNs and practical deployment issues.

**Table 3:** A summary of the ChestX-ray8 and LIDC-IDRI datasets used in this study.

Dataset Name	Class	Number of Images	Total
ChestX-ray8	Normal	1000	3000
	Pneumonia (benign)	1000	
	Nodule (malignant)	1000	
LIDC-IDRI	Malignant	1000	2000
	Benign	500	
	Normal	500	

**Table 4:** Resource requirements for different image sizes. The upward arrow indicates that the larger the number the better.

Image Size	Resources Consumed (GB)	Duration of Training (Hours)	Accuracy (%) ↑
1024 × 1024	4.23	3.24	92.80
448 × 448	3.16	2.32	92.00
224 × 224	2.45	1.45	85.00

**Table 5:** Accuracy and loss values for different epochs of a hybrid quantum model. The upward arrow indicates that the larger the number the better. The downward arrow indicates that the smaller the number the better.

Epochs	Accuracy (%) ↑	Loss (%) ↓
50	10.52	89.48
100	25.32	74.68
150	50.78	49.22
200	65.41	34.59
250	81.45	18.55
300	85.32	14.68
350	86.87	13.13
400	87.74	12.26
450	89.25	10.75
500	92.12	7.88
550	90.15	7.89

**Table 6:** Comparison of performance metrics between the system with and without the quantum classifier.

Model Name		Overall Accuracy (%)	Sensitivity (%)	Specificity (%)	F1-Score (%)	Precision (%)	MCC (%)
Traditional	VGG16	85.21	84	86	85	84	0.7
	VGG19	87.54	86	88	87	87	0.74
	InceptionV3	76.52	77	76	76	75	0.53
	Xception	74.25	75	74	74	73	0.48
	ResNet50	65.25	66	65	65	64	0.3
	RepVGG	89.21	89	90	89	89	0.78
Hybrid	VGG16	89.21	89	90	89	89	0.78
	VGG19	89.16	89	90	89	88	0.78
	InceptionV3	89.78	90	89	90	90	0.79
	Xception	85.23	85	86	85	84	0.7
	ResNet50	83.12	83	84	83	82	0.66
	RepVGG	79.45	80	79	79	78	0.58
		92.12	93	93	96	94	0.84

**Table 7:** Comparative analysis of misclassified cases.

System Type	Model Name	TP	TN	FP	FN
Traditional	VGG16	4050	200	450	300
	VGG19	4100	150	350	300
	InceptionV3	3500	200	1000	300
	Xception	3000	700	1000	300
	ResNet50	3000	200	1500	300
	RepVGG	4000	500	200	300
Hybrid	VGG16	4300	150	200	350
	VGG19	4200	250	200	300
	InceptionV3	4050	200	425	325
	Xception	4000	175	500	325
	ResNet50	3500	500	650	350
	RepVGG	4400	200	300	100

**Table 8:** Performance analysis of models with feature merging.

Model Name	Feature Dimension (Without Merging)	Accuracy without Merging (%)	Dimension after Fusion	Accuracy with Merging (%)
VGG16	512	84.5	1024	89.16
VGG19	512	85	1024	89.78
InceptionV3	2048	80.75	4096	85.23
Xception	2048	78.5	4096	83.12
ResNet50	2048	75	4096	79.45
RepVGG	2048	87.5	4096	92.12

**Table 9:** Comparison of our hybrid quantum system with other state-of-the-art systems. The upward arrow indicates that the larger the number the better. The downward arrow indicates that the smaller the number the better.

Technique	Accuracy (%) ↑	Computational Training Time (Hours) ↓
QCNN [27]	89.50	2.8
VQDNN [28]	90.00	2.52
Hybrid TL [29]	91.32	3.23
Quanvolution [31]	88.24	2.45
Proposed system	92.12	2.32

## Data Availability

The data supporting the findings of this study are available from the corresponding author upon reasonable request.
